# Model-based analysis of causes for habitat segregation in *Idotea* species (Crustacea, Isopoda)

**DOI:** 10.1007/s00227-016-2843-9

**Published:** 2016-03-14

**Authors:** Maximilian Strer, Arne Hammrich, Lars Gutow, Sylvia Moenickes

**Affiliations:** Institute of Land Use Systems, Leibniz Centre for Agricultural Landscape Research (ZALF), Eberswalder Straße 84, 15374 Müncheberg, Germany; DHI-WASY, Max-Planck-Str 6, 28857 Syke, Germany; Alfred Wegener Institute Helmholtz Centre for Polar and Marine Research, Am Handelshafen 12, 27570 Bremerhaven, Germany; Institut für Geoökologie, Technische Universität Braunschweig, 38106 Brunswick, Germany; Environmental Systems Analysis, Faculty of Life Sciences, Rhein-Waal University of Applied Sciences, 47533 Kleve, Germany

**Keywords:** Physiologically structured population model (PSPM), Intraguild predation, Competition, *Idotea balthica*, *Idotea granulosa*, Tidal influence on habitat segregation

## Abstract

On the shore of the rocky island of Helgoland (North Sea) two closely related isopod species, *Idotea balthica*Pallas, 1772, and *Idotea granulosa*Rathke, 1843, share a similar fundamental niche but inhabit well-separated habitats. *Idotea balthica *inhabits floating algae at the sea surface and accumulations of decaying algae on the seafloor, whereas *I. granulosa *primarily occurs in intertidal macroalgal belts. In laboratory experiments on individually reared isopods *I. balthica *outperformed *I. granulosa *with regard to growth, reproduction, and mortality in both a fully inundated habitat and in a tidal habitat with 5 h of daily emergence. We hypothesized that habitat segregation in the two isopod species is driven by one or multiple types of biotic interactions: (1) no interaction, (2) cannibalism, (3) intraguild predation, and (4) terrestrial predation. In order to evaluate how habitat segregation can be explained by each of these interaction types we employed a size-structured population model to account for the body-size-dependent predation. Net population growth rates were fitted to the simulations as a measure of population fitness. Experimental results served as database for parameter and process identification. As predation rates were unknown, we performed a sensitivity analysis for these. We found that below 5 h of daily tidal emergence either cannibalism or terrestrial predation sufficed to explain habitat segregation. Intraguild predation, in contrast, advantaged *I. balthica *in any case. From linear extrapolation of the effects occurring under conditions of 5 h of daily tidal emergence, we predict that contrasting physiological responses in *I. balthica *and *I. granulosa *would cause segregation even without any interaction if emergence lasted long enough.

**Electronic supplementary material:**

The online version of this article (doi:10.1007/s00227-016-2843-9) contains supplementary material, which is available to authorized users.

## Introduction

Habitat segregation among ecologically similar species is a common phenomenon for which several explanations have been proposed (Kroer 1986; Franke et al. 2006). A primary explanation suggests that species, which share the same fundamental niche, might slightly differ in their tolerances for prevailing environmental conditions (Brown and Feldmeth 1971). This resulted in a separation of the realized niches if one species is superior in competing for a common resource or in escaping from a common predator (Mac Arthur 1969; Schoener 1974; Morris 2003). Interspecific competition might be amplified by asymmetric intraguild predation where the predator benefits not only from the gain in assimilated energy but also from the weakening of a competitor (Polis et al. 1989; Arim and Marquet 2004). For example, threespine sticklebacks, *Gasterosteus aculeatus*, shift from benthic to pelagic food sources when exposed to intraguild predation by prickly sculpin, *Cottus asper* (Ingram et al. 2012). Hence, intraguild predation can support the establishment of two separate realized niches within one common fundamental niche (Wissinger 1992). Likewise, asymmetric predation by a common predator can lead to habitat segregation among species preyed upon (Schoener 1974). Seven marine isopod species of the genus *Idotea* inhabit the rocky shore of the island of Helgoland in the German Bight, North Sea. Although the two species *Idotea balthica *Pallas, 1772 and *Idotea granulosa *Rathke, 1843 co-occur on a broader geographic scale (Naylor 1955; Schmitt 1987; Healy and O’Neill 1984; Salemaa 1986; Borowsky 1987; Leidenberger et al. 2015), the habitats of these species are clearly segregated at a small spatial scale. *I. balthica *lives on floating seaweeds at the sea surface or in accumulations of macro-algal debris at the sea floor (Franke and Janke 1998; Vetter et al. 1999; Gutow et al. 2007), whereas *I. granulosa *inhabits intertidal macroalgae (Naylor 1955; Salemaa 1979; Salemaa and Ranta 1991; Leifsson 1998). However, the factors that shape this clear habitat segregation are as yet poorly understood.

The two model species considered in this study, *I. balthica* and *I. granulosa*, have very similar environmental requirements (Table 1). *Idotea granulosa *can cope with strong salinity fluctuations (Naylor 1955). Similarly, the euryhaline *I. balthica *tolerates salinities of 4–33 ‰  (Kroer 1986). Both species are found on floating macroalgae in the North Sea although *I. granulosa *is clearly a less common rafter than *I. balthica *(Franke et al. 1998; Gutow et al. 2015). Finally, both species are primarily herbivorous. However, substantial amounts of crustacean tissue have been found in the guts of *I. balthica *indicating that this species also uses animal food resources (Douglass et al. 2011). Accordingly, Kroer (1986) doubted that competition for food explains the absence of *I. balthica *from the intertidal zone. Alternatively, Franke and Janke (1998) suggested cannibalism and intraguild predation as proximate causes for habitat segregation in *Idotea* species. Therefore, we suggest that differential tolerance for tidal emergence as well as biotic interactions cause habitat segregation in *I. balthica *and *I. granulosa*. The aim of this paper is to analyse the potential of each of these factors to promote habitat segregation in the field.

**Table 1:** Characteristics of *Idotea* spp.

	*I. balthica*		*I. granulosa*	
Body length	10–18/30 mm	Naylor (1955)	6–13/20 mm	Salemaa (1979)
	10.2–13.5 mm*	Zaabar et al. (2015)		
Reproduction length	$12.8\pm 1.7$ mm	Kroer (1989)	$10.9\pm 1.6$ mm (min. 7.2 mm)	Leifsson (1998)
	6.8–8.6 mm*	Zaabar et al. (2015)		
Life span	$23\pm 11$ w	Gutow (2003)	1 year	Salemaa (1979)
	10–14 month*	Zaabar et al. (2014)		
Last reproduction	10 month*	Zaabar et al. (2015)		
Distribution	Worldwide	Borowsky (1987)	White Sea	Naylor (1955)
			North Sea	Healy and O’Neill (1984)
			Baltic Sea	Salemaa (1986)
Habitat	Floating macroalgae, sea floor	Franke and Janke (1998), Vetter et al. (1999)	Lower eulittoral	Naylor (1955), Salemaa (1979), Salemaa and Ranta (1991), Leifsson (1998)

Body lengths are specified for females/males, respectively

*Asterisk* refers to subspecies *Idotea balthica basteri*

Previous observations suggest four different scenarios of habitat segregation in the case of *Idotea* spp.: (1) Habitat segregation results from individual population response to environmental gradients (Rakocinski et al. 1992). Tidal gradient is a dominant environmental factor and has a strong influence on population composition (Kneib 1984; Troch et al. 2003) in the intertidal zone. (2) Habitat segregation results from fitness differences caused by both differentiation of the abiotic niche and cannibalism (Kneib 1984). (3) Habitat segregation results from both differentiation of the abiotic niche and intra- and inter-specific interference, including cannibalism and mutual predation (Franke et al. 2006). (4) Habitat segregation results from differentiation of the abiotic niche and differential predation by predators. Bell (1980) as well as Kneib and Stiven (1982) found that density of benthic invertebrates increases with the absence of predators in the intertidal zone.

For a quantitative analysis we assume that the species with the lower fitness will be out-competed over time. Thus, if the overall population fitness of both species can be quantified in terms of the net population growth rate (NPGR), the effects of each of the above four potential scenarios of habitat segregation can be estimated. Specifically, we can identify the parametric position at which both species have identical NPGR and, hence, share the same habitat—such a state is called tipping point according to Douglass et al. (2011). Since biotic interference is size dependent (Franke and Janke 1998; Leonardsson 2008) any population model that considers all mechanisms involved in any of the proposed processes must be a size-structured population model. Consequently, the NPGR must be determined inversely from direct simulations of population dynamics. Herein, we developed a size-structured population model including experimentally determined rates of individual growth, mortality, reproduction, and predation for both isopod species. A sensitivity analysis backed the *a priori* unknown parametrization of predation. We then determined NPGR and analysed the proposed drivers of habitat segregation for two different tidal regimes.

## Materials and methods

### Model species and experiments

Life history parameters of the two isopod species *I. balthica *and *I. granulosa *were determined from laboratory experiments which were conducted in walk-in climate rooms at the Helgoland Marine Station of the Alfred Wegener Institute. The isopods were taken from flow-through mass cultures (volume: 40 L) which were continuously supplied with animals from the field. The cultures were run at a constant temperature of 16 $^\circ$C and a light/dark rhythm of L:D = 16:8 h. Thalli of the brown algae *Fucus vesiculosus*L., 1753 and *Ascophyllum nodosum*Le Jolis, 1863 and nauplii of the brine shrimp *Artemia* spp. Leach, 1819 were offered as food to the isopods. The algae were collected from the rocky intertidal of the island of Helgoland. No tides were simulated in the cultures.

For the experiments, isopods of both species were reared individually for 21 weeks starting from the day of hatching from the mother’s marsupium. Each individual was kept in a glass vial (diameter: 60 mm; volume: 50 ml) at a constant temperature of 16 $^\circ$C and a light regime of L:D = 16:8 h. Fragments of *F. vesiculosus* and *Artemia* spp. nauplii were offered *ad libitum* as food. The seawater medium was exchanged daily for fresh, pre-tempered, sand-/gravel-filtered North Sea water.

Seventy-two individuals of each species were assigned to one of two different tidal treatments. In the control treatment without tidal emergence the isopods were permanently submerged. In the treatment with tidal emergence the seawater medium was removed each day at the same time for 5 h. In the course of the daily seawater exchange the isopods were monitored for survival and moulting. The sex of each individual was determined as soon as possible. Whenever an adult female was about to moult, a male from the same treatment group was added for 24 h for mating. When gravid females released offspring, the juveniles were counted. Body length of each individual was measured directly after moulting to the nearest millimetre using the distance from the frontal edge of the cephalon to the tip of the telson.

### Population model

In order to explicitly model the different scenarios, a size-structured population model was required, whereas the criterion chosen for a comparison of fitness, the NPGR, is meaningful for unstructured populations. After describing our structured population model we present our approach to deduce NPGR from its simulations.

Physiologically structured population models (de Roos 1997) take into account the distribution of determined physiological quantities within the population. Here, we take advantage of a size-structured population model in order to integrate size-specific predation. For each sex of each species and each environmental treatment, we have:

$$\begin{aligned} \frac{\partial u(t,l)}{\partial t}= \frac{\partial (g(l) \; u(t,l))}{\partial l}-m(t,l)\cdot u(l,t)+ r(t,l)-p(t,l) \cdot u(t,l) \end{aligned}$$

with the population distribution per body length *u*(*t*, *l*) [mm$^{-1}$] with time *t* [d] and length *l* [mm], individual growth *g*(*l*) [mm$\cdot$d$^{-1}$], mortality *m*(*t*, *l*) [d$^{-1}$], reproduction *r*(*t*, *l*) [mm$^{-1}$ d$^{-1}$] and predation *p*(*t*, *l*); the latter comprises cannibalism $p_{\text {C}}(t,l)$ [d$^{-1}$], competition $p_{\text {I}}(t,l)$ [d$^{-1}$], or terrestrial predation $p_{\text {T}}(t,l)$ [d$^{-1}$], respectively.

While reproduction, mortality, and all kinds of predation were defined straightforwardly, individual growth was based on the three different models including von Bertalanffy (Kozlowski et al. 2004), logistic growth (Strong and Daborn 1979), or logistic growth with a size-dependent growth rate (Table 2). With respect to first order mortality, we allowed for size-dependent mortality distinguishing basic and senile mortality. Reproduction was described based on two assumptions. First, we assumed that the per female reproduction *b*(*l*) is normally distributed over size such that integration over size of all females $u_\text {f}$ weighted by *b*(*l*) gives total reproduction. Second, we assumed that total reproduction exhibits normally distributed birth lengths.

**Table 2:** Process models and parameters

Process		Parameter		
*Individual growth*				
Von Bertalanffy	$g(l)= \rho \cdot ~(l_{\text {max}}-l)$	Maximal length	$l_{\text {max}}$	[mm]
Logistic growth	$g(l)= \rho \cdot ~l \cdot ~(1- \frac{l}{l_{\text {max}}})$	Critical length	$l_{\text {crit}}$	[mm]
Idem size dependent	$g(l)= \rho (l) \cdot l \cdot (1- \frac{l}{l_{\text {max}}})$	Growth rate	$\rho$	[1/d]
with		Juvenile growth rate	$\rho _{\text {j}}$	[1/d]
Growth rate	$\rho (l)=\rho _{\text {a}}-(\rho _{\text {a}}-\rho _{ \text {j}}) \cdot \exp {(-(\frac{l}{l_{\text {crit}}})^{\beta })}$	Adult growth rate	$\rho _{\text {a}}$	[1/d]
		Steepness	$\beta$	[–]
*Mortality*				
	$$m(l)={\left\{ \begin{array}{ll}\mu &{} ,l<l_{\text {s}}\\ \mu _{\text {s}} &{}, l \ge l_{\text {s}} \end{array}\right. }$$	Mortality rate	$\mu$	[1/d]
		Critical senile length	$\Delta _s$	[mm]
with	$l_{\text {s}} = l_{\text {max}}-\Delta _s$	senile mortality rate	$\mu _{\text {s}}$	[1/d]
*Reproduction*				
	$r(t,l)= \int _{0}^{\infty }b(l) u_{\text {f}} dl \cdot N(l,l_{\text {b}},\sigma _{\text {b}})$	Birth rate	$f_{\text {max}}$	[1/w]
with		Mean birth length	$l_{\text {b}}$	[mm]
Reprod. per female	$b(l) = f_{\text {max}} \cdot N(l,l_{\text {r}},\sigma _{\text {r}})$	Mean reproduction length	$l_{\text {r}}$	[mm]
		SD of birth length	$\sigma _{\text {b}}$	[mm]
		SD of reproduction length	$\sigma _{\text {r}}$	[mm]
		Normal distribution	$N(l,\bar{l},\sigma )$	[1/mm]
*Cannibalism*				
	$p_{\text {C}}(t,l)= -\upsilon \cdot \frac{\int {\alpha (l,\varLambda ) \cdot \varLambda ^3 \cdot P_{C}} ~d\varLambda }{\int {\varLambda ^3 \cdot P_{C}}~d\varLambda }$	Cannibalistic predation rate	$\upsilon$	[1/d]
		Minimum predator size	$\varLambda _\text {min}$	[mm]
with		Minimum size difference	$\Delta _{\text {min}}$	[mm]
		Threshold predator size	$\varLambda _\text {u}$	[mm]
Vulnerability	$$\alpha (\lambda ,\varLambda )= {\left\{ \begin{array}{ll} 0 &{} \varLambda \le \varLambda _\text {min}\\ 0 &{} \varLambda \le \lambda +\Delta _{\text {min}}\\ A(\lambda ,\varLambda _\text {u})&{} \varLambda _\text {u} < \varLambda \\ A(\lambda ,\varLambda ) &{} \text {else} \end{array}\right. }$$	Selective range	*S*	[mm]
Baseline vuln.	$A(\lambda ,\varLambda ) = \min (1,\frac{\varLambda -\lambda +\Delta _{\text {min}}}{S})$			
Predatory mass	$P_{C}=u_{\text {f}}+ u_{\text {m}}$			
*Intraguild predation*				
	$p_{\text {I}}(t,l)= -\varUpsilon \cdot \frac{\int {\alpha (l,\varLambda ) \cdot \varLambda ^3 \cdot P_{I}} ~d\varLambda }{\int {\varLambda ^3 \cdot P_{I}}~d\varLambda }$	Intraguild predation rate	$\varUpsilon$	[1/d]
with				
Predatory mass	$P_{I}=u_{\text {fv}}+u_{\text {mv}}+ u_{\text {fw}}+ u_{\text {mw}}$			
*Terrestrial predation*				
	$$p_{\text {T}}(t,l)={\left\{ \begin{array}{ll} 0,& l < l_{\text {P}} \\ -\tau ,& l \ge l_{\text {P}} \end{array}\right. }$$	Critical prey length	$l_{\text {P}}$	[mm]
		Terrestrial predation rate	$\tau$	[1/d]

Mortality, reproduction and predation were uniquely defined, growth was defined by a set of models subjected to model selection. Indices of population distribution per body length *u* signify $u_{\text {f}}$, female, $u_{\text {m}}$, male, $u_{\text {v}}$, species v, $u_{\text {w}}$, species w

For both kinds of interference predation, i.e. cannibalism and intraguild predation, we assumed predation to scale with the probability to interact with a predator. We determined that probability as the proportion of total predator volume in the total population volume through volume-weighted integrations. In doing so we assumed that the vulnerability of a prey varies with both its own body length $\lambda$ and that of the predator $\varLambda$ (Leonardsson 2008); hence, we weighted the integration of the predator volume over the predator body length with a length-specific vulnerability $\alpha (\lambda ,\varLambda )$. The vulnerability of a predator ranges between 1, if it is large enough to prey upon the other, and 0, if it is not (see electronic supplementary material for illustration). Finally, terrestrial predation was defined to be linear assuming a critical prey length $l_p$.

Integrating the resulting population distribution per body length *u*(*t*, *l*) over length *l* yields total population $U(t)=\int u(t,l) dl$. We assumed that the total population exhibits exponential dynamics, i.e. $U(t) \simeq U_{0} \exp {(k t)}$ with initial total population $U_{0}$ and NPGR *k* [1/d]. Hence, we identified NPGR through fitting the total population model to integrated structured population distributions *U*(*t*) (see electronic supplementary material for a detailed description).

### Parameter identification and model selection

Each of the process models were fitted to both experimental treatments for both species through the subspace trust region optimization algorithm implemented in MATLAB (MathWorks 2010), allowing for nonlinear optimization. The algorithm furnishes local optima only, so manual checking and repetition were applied where necessary.

Mortality and reproduction were defined straightforwardly from weekly averages of experimental data. In contrast, five different models were compiled for individual growth and subjected to model selection: von Bertalanffy (G1), logistic growth (G2), logistic growth with reference maximum length (G3), size-dependent logistic growth (G4), and size-dependent logistic growth with reference maximum length (G5). Akaike information criterion (Akaike 1974, AIC) was used as model selection criterion.

At first, models for individual growth *g*(*l*) were directly fitted to measurements $\frac{\hbox {d} l}{\hbox {d} t}=g(l)$. For fitting reproduction per female *b*(*l*) time-dependent data had to be transformed to size-dependent data. The transformation was defined through the respective optimal growth model. For mortality *m*(*t*, *l*) time- and size-dependent experimental data were used, and the dynamics of the experimental cohort *u*(*t*, *l*) were matched taking into account individual growth *g*(*l*) as given through the optimal growth model and *m*(*t*, *l*).

### Scenario analysis

For each scenario we combined the respectively selected processes and its parameterization to a full size-structured population model. As mean birth length $l_{\text {b}}$ and its standard deviation $\sigma _{\text {b}}$ had not been recorded in the experiment, we deliberately set them to 2 and 0.25 mm, respectively. Furthermore, predation rates $\upsilon$, $\varUpsilon$ and $\tau$ were unknown. Hence, we ran simulations for a range of rates. NPGR (i.e. *k*) was determined for both *I. balthica *and *I. granulosa *and for reference and tidal emergence treatments and compared as follows:

#### Scenario i: comparison for abiotic niche differentiation

Here, $p(l,t)=0$. If NPGR for *I. balthica *excels in the reference treatment and that for *I. granulosa *excels in the tidal treatment, then abiotic niche differentiation alone sufficiently explains habitat segregation above 5 h of tidal emergence.

#### Scenario ii, iii, and iv: capability analysis

For these scenarios, $p_\text {C}$, $p_\text {I}$, and $p_\text {T}$ were inserted as *p*(*l*, *t*), respectively. Here, the scenario reads: If a range of values of the predation rate $\upsilon$, $\varUpsilon$ or $\tau$ exists, for which NPGR for *I. balthica *excels in the reference treatment and that for *I. granulosa *excels in the tidal treatment, then the respective type of predation sufficiently explains habitat segregation above 5 h of tidal emergence.

### Gradient analysis

The experimental treatments allowed for parameterization and hence analysis of two distinct environmental conditions: full-time water coverage through the reference treatment and diurnal low water of $\Delta \theta = 5$ h through the tidal treatment. In order to simulate the population dynamics along the full gradient of tidal durations, we assumed linearity in all parameters. That is, we approximated the value of any parameter *p* at the tidal duration $\theta$ [h] following:

$$\begin{aligned} p(\theta )= \left( 1-\frac{\theta }{\Delta \theta }\right) p_{\text {ref}}+\frac{\theta }{\Delta \theta }p_{\text {tides}} \end{aligned}$$

with $p_{\text {ref}}$ and $p_{\text {tides}}$ being the estimated values for the reference and tidal treatment, respectively.

We simulated population dynamics (1) without predation, (2) including intraguild predation, (3) including terrestrial predation, and (4) including both. The respective predation rates were set to $\varUpsilon = 0.01$ d$^{-1}$ and $\tau (\theta )~=~0.01\text {(d h)}^{-1}\theta$. We determined NPGR for a range of tidal durations. From these, we finally determined the tidal duration at which the tipping point occurs.

## Results

### Parameter identification and process identification

**Fig. 1:**
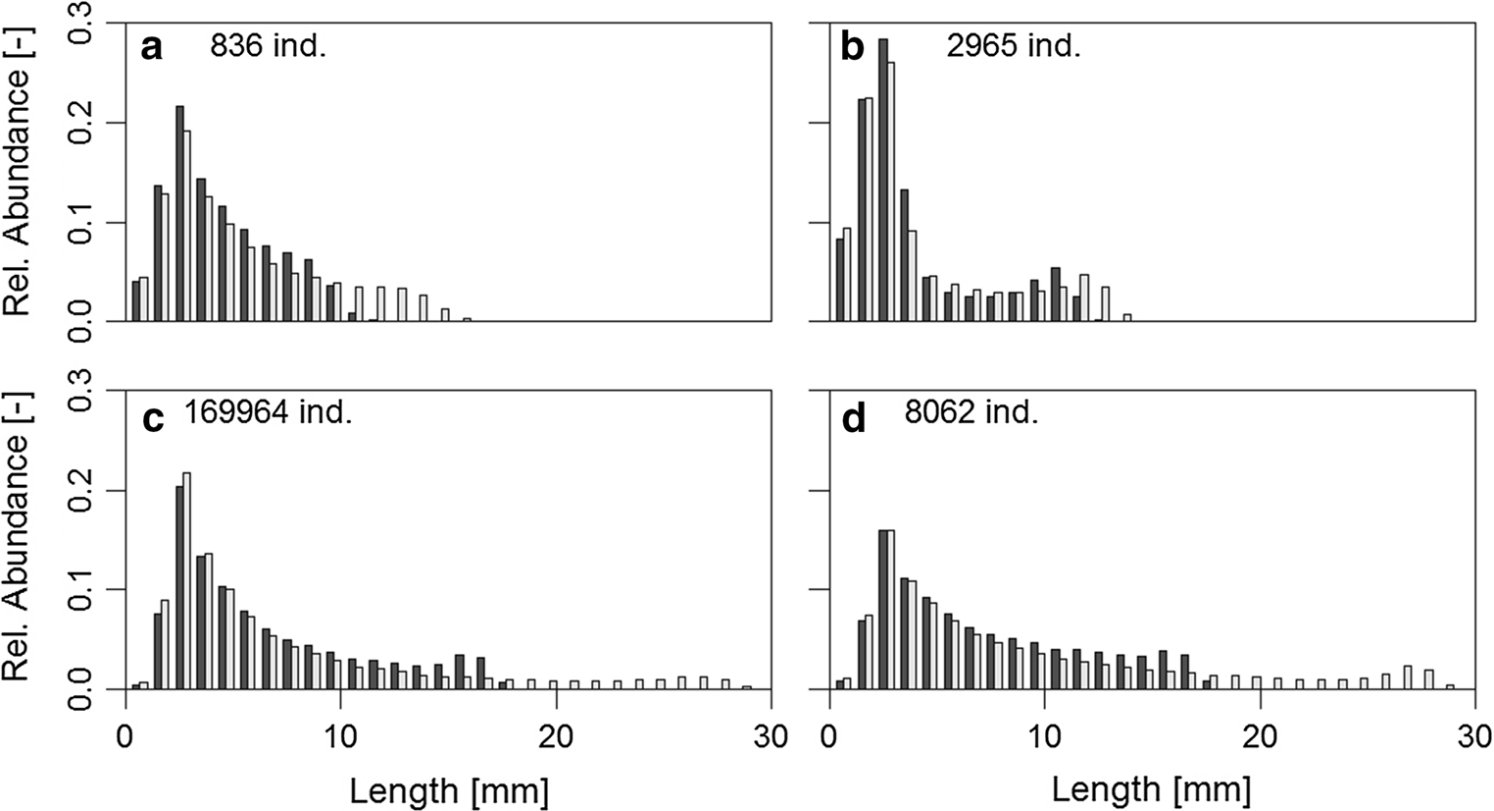
Population distribution according to the mechanisms of an abiotic niche (scenario i). Reference (**a**) and tidal emergence (**b**) of *I. granulosa *, and reference (**c**) and tidal emergence (**d**) of *I. balthica*. Distributions taken from simulations for estimation of NPGR (see electronic supplementary material); depicted are distributions at time *t* = 200 d. Relative abundances are shown, total abundances are given in the graphs. Male distribution in *light*, female distribution in *dark colours*, respectively

Parameter identification was feasible (Table 3) with highest coefficients of determination ($R^{2}$) above 0.9 growth and lowest for mortality of *I. balthica *(approximately 0.6). Process identification yielded logistic growth for each treatment. Determined maximum lengths ($l_{\text {max}}$) were similar to literature values (cf. Table 1). For *I. balthica *the maximum lengths were identical in both treatments, whereas they differed slightly between treatments for *I. granulosa*. Maximum length was larger in females that experienced tides, whereas for the males the maximum length was higher without tides (Fig. 1). Strong and Daborn (1979) found a logistic growth rate ($\rho$) for *I. balthica *males of 0.037 d$^{-1}$, i.e. of the same order of magnitude as our estimates. In the tidal treatment *I. granulosa *showed hampered juvenile growth (note model G4 in Table 3 and the differences in the shape of the population distribution in Fig. 1). Mean reproduction length of *I. granulosa *was similar to values reported by Leifsson (1998), whereas the mean reproductive length of *I. balthica *exceeded values reported by Kroer (1989).

**Table 3:** Parameter estimation and standard deviation for growth, mortality, reproduction, and NPGR for both species and both experimental treatments

Species treatment			*I. balthica*		*I. granulosa*	
			Reference	Tides	Reference	Tides
Individual growth			G2	G3	G2	G4
$l_{\text {max}}$	[mm]	f	$16.8\pm 0.2$	$16.8\pm 0.2$	$9.7\pm 0.3$	$10.8\pm 0.3$
		m	$27.8\pm 0.3$	$27.8\pm 0.3$	$14.6\pm 0.7$	$13.2\pm 0.3$
$\rho | \rho _{\text {a}}$	[1/d]	f	8.8E−02 ± 1.7E−03	6.7E−02 ± 1.1E−03	3.2E−02 ± 1.4E−02	8.2E−02 ± 1.4E−02
		m	7.3E−02 ± 7.3E−04	5.8E−02 ± 6.7E−04	2.7E−02 ± 3.9E−04	6.0E−02 ± 8.1E−03
$\rho _{\text {j}}$	[1/d]	f				1.7E−02 ± 2.8E−03
		m				1.3E−02 ± 6.5E−03
$l_{\text {crit}}$	[mm]	f				3.8 ± 0.5
		m				4.4 ± 0.7
$R^2$	[–]	f	0.95	0.97	0.95	0.94
		m	0.98	0.98	0.91	0.92
*Mortality*						
$\mu$	[1/d]	f	6.4E−04 ± 7.7E−05	2.9E−03 ± 2.5E−04	5.7E−03 ± 3.6E−04	6.5E−03 ± 3.5E−04
		m	6.4E−04 ± 7.7E−05	1.4E−03 ± 9.2E−05	2.1E−03 ± 2.5E−04	5.3E−03 ± 1.1E−04
$R^2$	[–]	f	0.58	0.48	0.93	0.88
		m	0.66	0.68	0.92	0.92
*Reproduction*						
$f_{\text {max}}$	[1/w]	f	$21\pm 3.71$	$14.9\pm 2.17$	$5.46\pm 0.16$	$11.7\pm 0.13$
$l_{\text {r}}$	[mm]	f	$16.3\pm 0.08$	$16.7\pm 0.08$	$8.43\pm 0.13$	$9.94\pm 0.11$
$\sigma _{\text {r}}$	[mm]	f	$0.39\pm 0.08$	$0.64\pm 0.12$	$0.8\pm 0.16$	$1.54\pm 0.13$
$R^2$	[–]	f	0.65	0.88	0.76	0.94
*Population growth*						
*k*	[1/w]	f	4.2E−01 ± 1.6E−03	3.0E−01 ± 1.6E−03	1.5E−01 ± 2.1E−03	1.7E−01 ± 1.8E−03

For individual growth, parameters are given for the selected model only (see indication)

Irrespective of the parameter measured, *I. balthica *always performed worse when experiencing tidal emergence. Accordingly, in our simulations of the full population dynamics for the abiotic niche the abundance of *I. balthica *in the reference exceeds that in the tidal treatment (Fig. 1). *I. granulosa*, in contrast, did not show a clear trend in its responses to the treatment. However, the abundance of this species was always higher in the tidal treatment than in the reference. Nevertheless, the simulated abundance was consistently higher in *I. balthica *than *I. granulosa *in both treatments.

### Analysis of scenarios

The competitiveness of the *Idotea* spp. is indicated by NPGR for each of the four habitat segregation scenarios. This rate is fitted to simulation results of each scenario, and each species. We comprise the following results for the segregation scenarios.

1. Differentiation of the abiotic niche
  The trends of NPGR (Table 3) agreed with those of the simulated abundances described in the previous section. Thus, NPGR of *I. granulosa* for the tidal treatment exceeded that of the reference treatment. In contrast, the NPGR of *I. balthica *was higher in the reference treatment than in the tidal treatment. Hence, *I. granulosa *took advantage of tidal emergence, whereas *I. balthica *suffered from regular emergence. Nevertheless, the NPGR was higher in *I. balthica *than in *I. granulosa *in both treatments, and scenario (i) does not explain habitat segregation.
2. Niche differentiation comprising intraspecific interference
  In both treatments the NPGR of *I. balthica *decreased with increasing cannibalism, whereas the NPGR of *I. granulosa *remained stable (see Fig. 2): The cannibalism model assumed a minimum length for a predator which was not reached by *I. granulosa*.
  In the tidal treatment the tipping point for the NPGR was reached at a predation rate ($\upsilon _\text {tides}$) at which the tipping point was not yet reached in the reference treatment ($\upsilon _\text {ref}>\upsilon _\text {tides}$). Clearly, if the cannibalistic predation rate assumes a value that lies between $\upsilon _\text {tides}$ and $\upsilon _\text {ref}$, then scenario (ii) sufficiently explains habitat segregation.

**Fig. 2:**
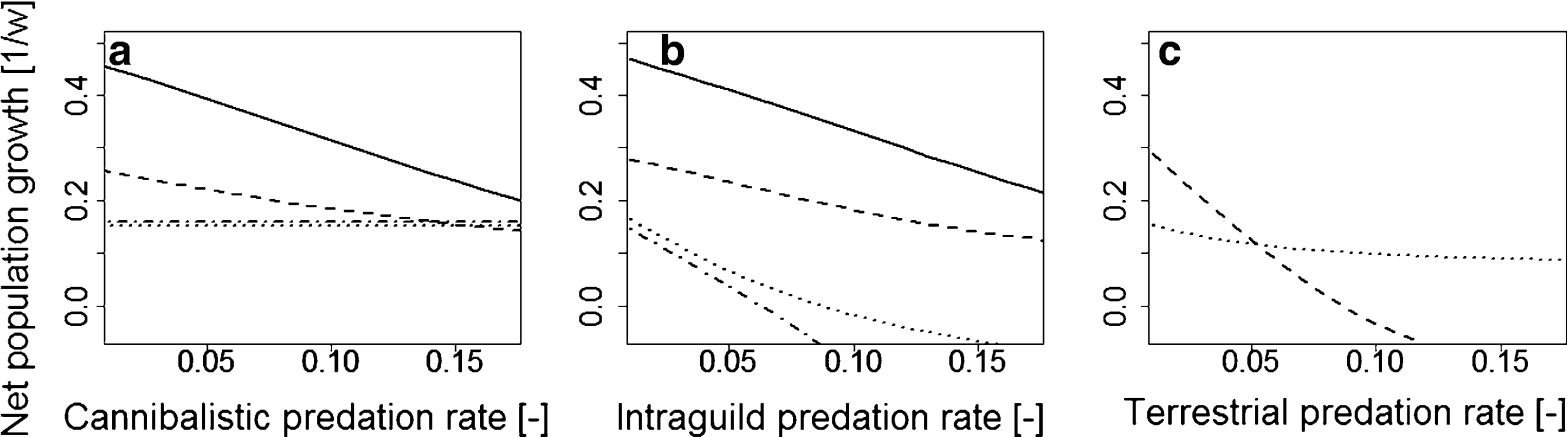
NPGR as depending on the strength of biotic interaction. **a** cannibalism (rate $p_{\text {C}}$), **b** intraguild predation (rate $p_{\text {I}}$), **c** terrestrial predation (rate $p_{\text {T}}$). *Solid lines* represent the reference treatment of *I. balthica *, *dashed lines* represents tidal treatment of *I. balthica*, *dash-dotted lines* represent reference treatment of *I. granulosa*, and *dotted lines* represent tidal treatment of *I. granulosa*
3. Competitive exclusion comprising intra- and interspecific interference
  All NPGR decreased with increasing intraguild predation (Fig. 2). More specifically, the effect of *I. granulosa *on *I. balthica *was small as the predator size was barely reached (compare previous scenario). In contrast, the effect of *I. balthica *on *I. granulosa *was large. *I. granulosa *was preyed upon over the full range of sizes such that its NPGR turned negative even at small predation rates and no tipping point existed for positive growth rates. Hence, scenario (iii) does not explain habitat segregation.
4. Niche differentiation comprising differential predation by terrestrial predators
  In the tidal treatment the NPGR decreased more strongly in *I. balthica* than in *I. granulosa* so that a tipping point was reached for positive growth rates (Fig. 2). This situation was mainly induced by differences in the mean reproduction length. *I. granulosa *reproduced below the minimum length for terrestrial predation, whereas *I. balthica *did not, so that reproduction of *I. balthica *was severely reduced. Hence, scenario (iv) does explain habitat segregation.

### Gradient analysis

Irrespective of the predation scenario, a tipping point could be identified along the tidal gradient (Table 4). The sensitivity of these points to predation rates was small.

**Table 4:** Tidal duration at which a tipping point occurs for reference parameterization of predation rates $p_0$ and $p_0 \pm$ 10 %

Predation scenario	Critical tidal duration $\theta$ [h]		
	$0.9 p_0$	$p_0$	$1.1 p_0$
None	–	8.1	–
Intraguild	7.0	7.0	6.9
Terrestrial	3.8	3.7	3.6
Intraguild and terrestrial	3.2	2.9	2.8

Remarkably, intraguild predation led to a negative NPGR in the inferior *I. granulosa *at a tidal duration of 5 h whereas it led to a tipping point at only moderately longer tidal emergence. Furthermore, the NPGR was strongly reduced when intraguild predation was combined with terrestrial predation which mainly affects larger individuals and hence reduces predation by *I. balthica*.

## Discussion

Habitat segregation in *Idotea* spp. in the tidal zone of Helgoland can be caused by various mechanisms. We tested four different explanations for habitat segregation in *Idotea balthica *and *I. granulosa *and could not clearly reject any of them. Given sufficient duration of regular tidal emergence, differences in specific physiological response to this environmental factor alone is able to cause a switch in predominance of either of the two species. When combined with intraguild predation or differential terrestrial predation or both, even shorter durations of tidal emergence result in such a switch. Thus, the shorter the tidal duration at which habitat segregation occurs, the higher is the probability that these interactive processes are involved in the generation and maintenance of habitat segregation in these two isopod species. A more detailed analysis of the relative importance of each process will require both a more detailed mathematical description and parametrisation of predation. In this study our experimental backing was limited to direct physiological response.

Especially for terrestrial predation, a mathematical description that is more accurate than ours and that does not advantage *I. granulosa*, is urgently needed. Specific field monitoring in combination with predator exclusion experiments could provide more detailed information on the mechanisms and actual importance of terrestrial predation. Intraguild predation was described following observations on other isopods, e.g. *Saduria entomon* (Leonardsson 2008) and from mechanisms described for cannibalism in size-structured population models (Cushing 1992). *Saduria entomon* is more carnivorous than *Idotea* spp., has a higher life expectancy and grows larger. Accordingly, applying these parameters to our model organisms favours the numerical dominance of *I. balthica *over *I. granulosa*. Additionally, we introduced intraguild predation only as supplemental source of mortality for the prey assuming that food is not limited. Under conditions of food limitation the predator certainly benefits from the energy gained from the assimilation of the prey (Polis et al. 1989; Wissinger 1992; Holt and Polis 1997).

Reduced juvenile growth in *I. granulosa *under the influence of tidal emergence also occurred under conditions of food limitation (Hammrich, unpublished). Furthermore, early reproduction of *I. granulosa *and the production of relatively large offspring (Salemaa 1979; Leifsson 1998) comply with typical reproductive strategies of *Idotea* spp. under high predation pressure (Tuomi et al. 1988). In the field, further environmental conditions do affect the species’ fitness and they may interact in various ways with the direct physiological response and indirect biotic effects. For instance, changes in ambient temperatures may influence life expectancy (Zaabar et al. 2014), or predator performance (Sanford 2002) but leave tolerance to tides less affected.

Our modelling approach can be summarized as a physiologically structured population model delivering exponential net population growth rates along a temporal gradient of tidal emergence. The structured population model offered a way to integrate size-specific predation and proved to be valid. For instance, it yielded a stable size distribution comparable to the results from other models (de Roos and Persson 2001; Cushing 1992). The assumption of exponential population growth is valid under ideal conditions but hardly ever realized under natural field conditions. Franke and Janke (1998) found that *I. balthica *and *Idotea emarginata* do reach limiting population capacities in a spatially restricted habitat. Finally, linearity in all parameters along a tidal gradient is an over-simplification. The specific parameters involved in the different mechanisms will vary in their sensitivity to tidal duration. Repetition of the experimental studies used in this model for parameter identification on predation is indispensable for further quantitative analysis of our research question.

In summary, the results of our modelling approach indicate that both species-specific physiological reactions including growth, mortality, and reproduction, and inter-specific interactions including competition, intraguild predation, and terrestrial predation contribute to the segregation of habitats. This habitat segregation of the two isopod species *I. balthica *and *I. granulosa *on the small scale allows for stable co-occurence on a larger spatial scale.

Our modelling approach involved data collected under highly controlled laboratory conditions. Accordingly, a considerable fraction of natural environmental variability was neglected probably inducing a certain bias in the model outcome. Additionally, predation was not quantified explicitly. Instead, its relevance for habitat segregation in *I. balthica *and *I. granulosa *was estimated from theoretical simulations. Intraguild predation is an important factor inducing habitat segregation in *Idotea* spp. at Helgoland (Franke and Janke 1998; Franke et al. 2006). Similarly, intense terrestrial predation can be expected from the abundant and diverse avifauna at this rocky island (Hüppop and Hüppop 2011). Manipulative field experiments are certainly needed to more accurately estimate the role of predation on the structuring of littoral isopod populations and to critically test the predictions derived from our model simulations.

## Electronic supplementary material

Below is the link to the electronic supplementary material.

Supplementary material 1 (pdf 172 KB)

Supplementary material 2 (pdf 106 KB)
