# Supplementary material for: Model-based analysis of causes for habitat segregation in Idotea species (Crustacea, Isopoda)
Source: Mar Biol. 2016 Mar 14;163:68. doi: 10.1007/s00227-016-2843-9 (PMC4789302; doi:10.1007/s00227-016-2843-9)
Supplement: Supplementary file 1 — Supplementary material 1 (pdf 172 KB) [file 227_2016_2843_MOESM1_ESM.pdf]

Marine Biology manuscript No.
(will be inserted by the editor)

Model-based analysis of causes for habitat segregation in *Idotea* (Crustacea, Isopoda) species

Supplementary material

Maximilian Strer · Arne Hammrich · Lars Gutow · Sylvia Moenickes

the date of receipt and acceptance should be inserted later

Size dependent vulnerability $\alpha(\lambda, \Lambda)$

In accordance with experimental observations of Leonardsson (2008) we define the vulnerability α to be dependent on the predator size Λ and the prey size λ. With the minimum predator size Λ_{min} [mm], minimum size difference Δ_{min} [mm], threshold predator size Λ_{u} [mm] and selective range S [mm] it reads

$$\alpha(\lambda, \Lambda) = \begin{cases} 0 & \Lambda \leq \Lambda_{\mathrm{min}} \\ 0 & \Lambda_{\mathrm{min}} < \Lambda \leq \Lambda_{\mathrm{u}} \ \& \ \Lambda \leq \lambda + \Delta_{\mathrm{min}} \\ \min(1, \frac{\Lambda - \lambda + \Delta_{\mathrm{min}}}{S}) & \Lambda_{\mathrm{min}} < \Lambda \leq \Lambda_{\mathrm{u}} \ \& \ \Lambda > \lambda + \Delta_{\mathrm{min}} \\ \alpha(\lambda, \Lambda_{\mathrm{u}}) & \Lambda_{\mathrm{u}} < \Lambda \end{cases}$$

Figure 1 Size dependent vulnerability model $\alpha(\lambda, \Lambda)$, predator of size Λ excert distinct predation pressure on the prey of size λ

Determination of NPGR k

At first, we simulated the size-structured population distribution per body length $u(t, l)$ for each sub-population. Based on the original experiments the initial distribution consists of 36 individuals uniformly distributed around the mean birth length of $l_{\mathrm{b}} = 2$ mm and the standard deviation of birth length $\sigma_{\mathrm{b}} = 0.25$ mm. The simulation time was 200 days in order to allow populations to develop a stable size-structure. The simulated population distribution $u(t, l)$ was then integrated over length $U(t) = \int u(t, l) dl$ in weekly time intervals. On these data the NPGR k was estimated through the subspace trust region optimisation algorithm implemented in Matlab (MathWorks 2010), allowing for non-linear optimisation. The algorithm furnishes local optima only and manual checking and repetition was applied where necessary.

References

Leonardsson K (2008) Effects of cannibalism and alternative prey on population dynamics of *Saduria entomon* (Isopoda). Ecol 72(4):1273–1285

MathWorks (2010) Matlab R2010. MathWorks Inc.
