# Supplementary material for: Model-based analysis of causes for habitat segregation in Idotea species (Crustacea, Isopoda)
Source: Mar Biol. 2016 Mar 14;163:68. doi: 10.1007/s00227-016-2843-9 (PMC4789302; doi:10.1007/s00227-016-2843-9)
Supplement: Supplementary file 2 — Supplementary material 2 (pdf 106 KB) [file 227_2016_2843_MOESM2_ESM.pdf]

Marine Biology manuscript No.
(will be inserted by the editor)

Model-based analysis of causes for habitat segregation in *Idotea* species (Crustacea, Isopoda)

Supplementary Material

Maximilian Strer · Arne Hammrich · Lars Gutow · Sylvia Moenickes

the date of receipt and acceptance should be inserted later

M. Strer

Institute of Land Use Systems, Leibniz Centre for Agricultural Landscape Research (ZALF), Eberswalder StraÃŸe 84, 15374 Müncheberg, Germany;

Previous address: Institut für Geoökologie, Technische Universität Braunschweig, 38106 Braunschweig, Germany

E-mail: Maximilian.Strer@zalf.de

A. Hammrich

DHI-WASY, Max-Planck-Str 6, 28857 Syke, Germany;

Previous address: Alfred-Wegener-Institut, Helmholtz Centre for Polar and Marine Research, Biologische Anstalt Helgoland, Postfach 180, Helgoland 27483, Germany

E-mail: arh@dhigroup.com

L. Gutow

Alfred Wegener Institute Helmholtz Centre for Polar and Marine Research, Am Handelshafen 12, 27570 Bremerhaven, Germany

E-mail: lars.gutow@awi.de

S. Moenickes

Environmental systems analysis, Faculty of Life Sciences, Rhein-Waal University of Applied Sciences, 47533 Kleve, Germany;

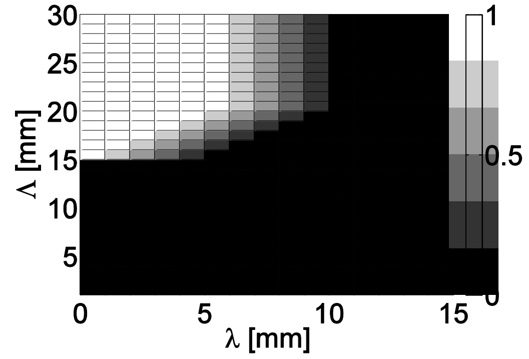

Figure 1: Size dependent vulnerability model $\alpha(\lambda, \Lambda)$, predator of size Λ excert distinct predation pressure on the prey of size λ

Previous address: Institut für Geoökologie, Technische Universität Braunschweig, 38106 Braunschweig, Germany

E-mail: sylvia.moenickes@hochschule-rhein-waal.de
